# Delayed Onset of Inhibition of Return in Visual Snow Syndrome

**DOI:** 10.3389/fneur.2021.738599

**Published:** 2021-09-17

**Authors:** Paige J. Foletta, Meaghan Clough, Allison M. McKendrick, Emma J. Solly, Owen B. White, Joanne Fielding

**Affiliations:** ^1^Department of Neuroscience, Central Clinical School, Monash University, Melbourne, VIC, Australia; ^2^Department of Optometry and Vision Sciences, The University of Melbourne, Melbourne, VIC, Australia

**Keywords:** visual snow, visual snow syndrome, visual processing, attention, inhibition of return, ocular motor

## Abstract

Visual snow syndrome (VSS) is a complex, sensory processing disorder. We have previously shown that visual processing changes manifest in significantly faster eye movements toward a suddenly appearing visual stimulus and difficulty inhibiting an eye movement toward a non-target visual stimulus. We propose that these changes reflect poor attentional control and occur whether attention is directed exogenously by a suddenly appearing event, or endogenously as a function of manipulating expectation surrounding an upcoming event. Irrespective of how attention is captured, competing facilitatory and inhibitory processes prioritise sensory information that is important to us, filtering out that which is irrelevant. A well-known feature of this conflict is the alteration to behaviour that accompanies variation in the temporal relationship between competing sensory events that manipulate facilitatory and inhibitory processes. A classic example of this is the “Inhibition of Return” (IOR) phenomenon that describes the relative slowing of a response to a validly cued location compared to invalidly cued location with longer cue/target intervals. This study explored temporal changes in the allocation of attention using an ocular motor version of Posner's IOR paradigm, manipulating attention exogenously by varying the temporal relationship between a non-predictive visual cue and target stimulus. Forty participants with VSS (20 with migraine) and 20 controls participated. Saccades were generated to both validly cued and invalidly cued targets with 67, 150, 300, and 500 ms cue/target intervals. VSS participants demonstrated delayed onset of IOR. Unlike controls, who exhibited IOR with 300 and 500 ms cue/target intervals, VSS participants only exhibited IOR with 500 ms cue/target intervals. These findings provide further evidence that attention is impacted in VSS, manifesting in a distinct saccadic behavioural profile, and delayed onset of IOR. Whether IOR is perceived as the build-up of an inhibitory bias against returning attention to an already inspected location or a consequence of a stronger attentional orienting response elicited by the cue, our results are consistent with the proposal that in VSS, a shift of attention elicits a stronger increase in saccade-related activity than healthy controls. This work provides a more refined saccadic behavioural profile of VSS that can be interrogated further using sophisticated neuroimaging techniques and may, in combination with other saccadic markers, be used to monitor the efficacy of any future treatments.

## Introduction

Visual Snow Syndrome (VSS) is a complex sensory disorder that diagnostically manifests in a range of debilitating visual symptoms, at its core, a persistent positive visual disturbance known as visual snow (1). While not included among the syndrome's diagnostic criteria, VSS participants often also experience other non-visual sensory changes, like tinnitus, migraine, paraesthesia, and depersonalisation. Unfortunately, there are no effective treatments for VSS, largely a consequence of an unknown aetiology. Current theories propose either widespread dysfunction of higher order visual processing areas (2–6), or direct thalamic dysfunction (7), although, as yet, the body of research conducted is small and inconclusive.

With the aim to provide objective evidence of neuropathological changes in VSS, our previous studies have investigated visual processing performance in VSS individuals using highly sensitive ocular motor (OM) tasks (8, 9). The first of these studies (8) demonstrated that participants with VSS generated faster eye movements toward suddenly appearing visual stimuli and failed more often to inhibit erroneous eye movements to stimuli not consistent with task demands. We also showed that this occurred irrespective of the complexity of the task presented. While we attributed this pattern of response to alterations in the early processing of visual stimuli within the visual regions of the cortex, we subsequently proposed that these results might also be interpreted as a more rapid shift of attention.

Our second study (9) sought to determine whether volitional shifts of attention elicited by a cue (endogenous driven shifts of attention), were similarly impaired, and revealed that VSS participants again failed more often to inhibit erroneous eye movements toward non-target locations. This demonstrated that attentional changes are evident in VSS irrespective of whether attention is directed exogenously by a suddenly appearing stimulus, or endogenously by manipulating expectation surrounding an upcoming event. We concluded that both exogenous and endogenous shifts of attention more strongly increase saccade-related activity in VSS, affecting the fine balance between saccade facilitation and inhibition, and manifesting as increased erroneous release of saccades to task irrelevant locations (increased errors) and altered saccade latency profiles.

Importantly, recent evidence has provided support for our supposition, with disruption reported in several cortical regions involved in the control of attention. For example, neuroimaging studies in VSS have found changes in grey matter volumes and reduced Blood Oxygenation Level-Dependent (BOLD) responses to visual stimulation similar to VS in several brain regions involved in attentional orienting, including the supramarginal gyrus and frontal eye fields (10). Widespread disruption has also been revealed in the functional connectivity of several brain systems, including attentional networks (5, 11). White matter abnormalities have been reported in the temporo-parieto-occipital junction in pathways related to vision (12). However, it is still unclear whether the attentional changes within the visual system indeed affect the balance between saccade facilitation and inhibition, in turn affecting the timing of saccade latencies.

Here we explored temporal changes in the allocation of attention in participants with VSS using a classic Posner style spatial cueing paradigm (13). This paradigm manipulates attention exogenously by varying the temporal relationship between a non-predictive visual cue and a target stimulus. Specifically, a cue, such a peripheral flash or change in luminance is presented prior to the presentation of a peripheral target, either in the same location as the target (valid cue) or in a different location to the target (invalid cue). Thus, the peripheral cue orientates attention to the cued location prior to the onset of a target. However, the effect of the cue varies as a function of the temporal relationship between the cue and target, or the stimulus onset asynchrony (SOA). Where there is a short delay between cue and target, the cue facilitates a subsequent response toward that location and delays a subsequent response away from that location. However, where there is a longer delay between cue and target, this relationship is reversed with a slower response to a target presented in the same location as the previously presented cue relative to a target presented elsewhere. The relative slowing of a response to a validly cued location is known as inhibition of return (IOR) and is attributed to the build-up of an inhibitory bias against returning attention to an already inspected location (14).

Disruption to neural function has been shown to alter the timepoint at which IOR occurs (i.e., transition from facilitation to inhibition for a validly cued trial). For example, using a modified ocular motor version of Posner's spatial cueing paradigm, Larrison-Faucher et al. (15) found a delay in the onset of IOR for patients diagnosed with schizophrenia. The authors attributed this to a delay in the build-up of inhibition toward the cued location. Conversely, Fielding et al. (16) reported accelerated onset of IOR in a group of patients with Huntington's disease. This was attributed to the altered inhibitory output of the basal ganglia and the premature disengagement (or removal of facilitatory activity) from a cued location.

We presented VSS participants with an ocular motor version of the spatial cueing paradigm to assess the time-course of IOR. Given that we have previously demonstrated stronger attentional capture by suddenly appearing stimuli in VSS participants, we anticipated that the transition to IOR might be delayed, a consequence of a stronger facilitatory effect of the cue, and that VSS participants would generate more erroneous saccades to cue stimuli. We propose that clarifying changes in the exogenous orienting of attention in VSS participants will enable us to develop a more refined objective behavioural marker of VSS that may be used to guide future research into mechanisms of dysfunction or as an objective outcome measure in treatment trials.

## Method

### Participants

Forty participants meeting the criteria for VSS as specified by the International Classification of Headache Disorders (ICHD: see Table 1) were recruited through a combination of online, radio and television advertising. Equivalent numbers of VSS participants with and without a history of migraine enabled us to determine whether any behavioural changes revealed in VSS participants were attributable to the presence of migraine. Of those with a history of migraine, none reported experiencing a migraine and/or migraine aura in the 3 days prior to or following testing. However, a series of analyses of variance (ANOVAs) between VSS participants with and without migraine revealed no significant differences between groups for any experimental variable (Table 2); consequently, data for all VSS participants were combined into a single group.

**Table 1:** International classification of headache disorders (ICHD-3) criteria for a diagnosis of visual snow syndrome.

A	Visual snow: dynamic, continuous, tiny dots across the entire visual field persisting for > 3 months
B	Additional visual symptoms of at least two of the following four types:
	i. Palinopsia. ii. Enhanced entoptic phenomena. iii. Photophobia iv. Nyctalopia (impaired night vision)
C	Symptoms are not consistent with typical migraine visual aura
D	Symptoms are not better accounted for by another disorder

**Table 2:** Demographic information for all participants.

	**VSS *Mean* (SD)** ***n* = 20**	**VSS + Migraine** ***Mean* (SD) *n* = 20**	**Controls *Mean* (SD)** ***n* = 20**
Female/male	9/11	15/5	13/7
Age/distribution	25.35/16–54	28.05/20–50	25.60/15–51
Visual snow			
Duration (years)	16.69 (13.34)	14.90 (12.29)	
Participants with lifelong duration (%)	55	45	
Afterimages (%)	83.3	88.9	
Photophobia (%)	88.9	83.3	
Nyctalopia (%)	77.8	61.1	
Floaters (%)	72.2	88.9	
Blue field entoptic phenomenon (%)	55.6	72.2	
Tinnitus (%)	55.6	66.7	
Paraesthesia (%)	33.3	33.3	
Family history of migraine	44.4	55.6	
Relative with VS (%)	0	5.6	
DASS			
Depression	8.78 (9.84)	8.28 (8.02)	3.06 (4.15)
Anxiety	5.72 (5.29)	6.50 (5.60)	3.65 (3.97)
Stress	13.17 (9.56)	11.61 (9.11)	6.82 (6.28)
AUDIT	5.06 (5.18)	4.72 (4.57)	3.00 (2.35)
DUDIT	0.5 (1.25)	1.89 (4.51)	0.42 (1.28)
FSS	36.5 (12.20)	39.72 (12.21)	29.06 (10.22)
NART	115.17 (5.26)	114.26 (5.07)	115.75 (7.22)

*DASS, Depression Anxiety Stress Scale; AUDIT, Alcohol Use Disorders Identification Test; DUDIT, Drug Use Disorders Identification test; FSS, Fatigue Severity Scale; NART, National Adult Reading Test*.

All VSS participants underwent a full ophthalmological examination to exclude any visual processing deficit. This involved an assessment of visual acuity, colour vison and retinal anatomy and function. Twenty neurologically healthy controls were recruited from the community. None reported a history of migraine. Exclusion criteria for all participants was the presence of a confounding neurological condition or the use of medication likely to affect vision or cognitive function.

All participants were asked to complete on online battery of questionnaires; 4 participants with VSS failed to fully complete the battery (two with migraine and two without) as did three healthy controls. The Alcohol Use Disorders Identification Test [AUDIT; (17)] and the Drug Use Disorders Identification test [DUDIT; (18)] were used to identify any substance abuse problems; scores on these measures did not differ significantly between controls and VSS participants. The National Adult Reading Test [NART; (19)] provided an estimate of intelligence. Again, no differences were revealed between groups. However, VSS participants scored higher on the Fatigue Severity Scale [FSS; (20)] [*F*_(1,51)_ = 7.01, *p* = 0.010], and the Depression Anxiety Stress Scale [DASS; (21)] over the past week; levels of depressive symptomology [*F*_(1,51)_ = 5.84, *p* = 0.019] and stress [*F*_(1,51)_ = 5.05, *p* = 0.029].

Table 2 provides a summary of demographic information for all participants, including a prevalence of commonly co-occurring visual symptoms associated with VSS.

### Procedure

All testing was conducted at the Central Clinical School in the Alfred Centre, Monash University, Australia. Ethical approval was granted by Monash University Human Research Ethics Committee. All participants provided informed consent prior to inclusion in the study in accordance with the Declaration of Helsinki.

#### Ocular Motor Spatial Cueing Task

Participants were seated in a darkened, quiet room, on a height adjustable chair in front of a monitor at a distance of 950 mm. A head and chin rest maximised head stability during recording. Displacement of the eye was recorded using an Eyelink 1,000+ dark pupil video-oculography system, which features high resolution (noise limited at <0.01°), and a high acquisition rate (1,000 Hz). Task stimuli comprised a white centrally located fixation cross (17 × 17 mm) on a black background with two white boxes (34 × 34 mm) situated eight degrees left and right of fixation. Green target crosses (25.5 × 25.5mm) were presented in the centre of one of the two white boxes.

The task used was a modified version of Posner and Cohen's (22) IOR paradigm. In total, the task comprised 246 randomly presented trials with breaks to mitigate fatigue. Participants were required to fixate on a central cross. Following 850 ms, one of the two peripheral boxes was illuminated for 50 ms. Participants were instructed to ignore this event and to maintain their gaze on the central cross. Following a variable delay of 17, 100, 250, or 450 ms, the central cross disappeared, and a green target cross appeared in either of the two peripheral boxes. This resulted in four stimulus onset asynchronies (SOAs); 67, 150, 250, and 500 ms, as used previously in saccadic IOR tasks (23). Participants were instructed to make an eye movement toward the target cross as soon as it appeared. Following 1,500 ms, gaze was reoriented back to centre by the presentation a small white square in preparation for the next trial.

Three trial types were included, determined by the relative location of the cue and target.

- Valid trial—cue and target presented in the same hemifield
- Invalid trial—cue presented in the hemifield opposite to the target
- Catch trial—cue presented but with no subsequent target; to reduce the likelihood of anticipatory responses.

**Figure 1:**
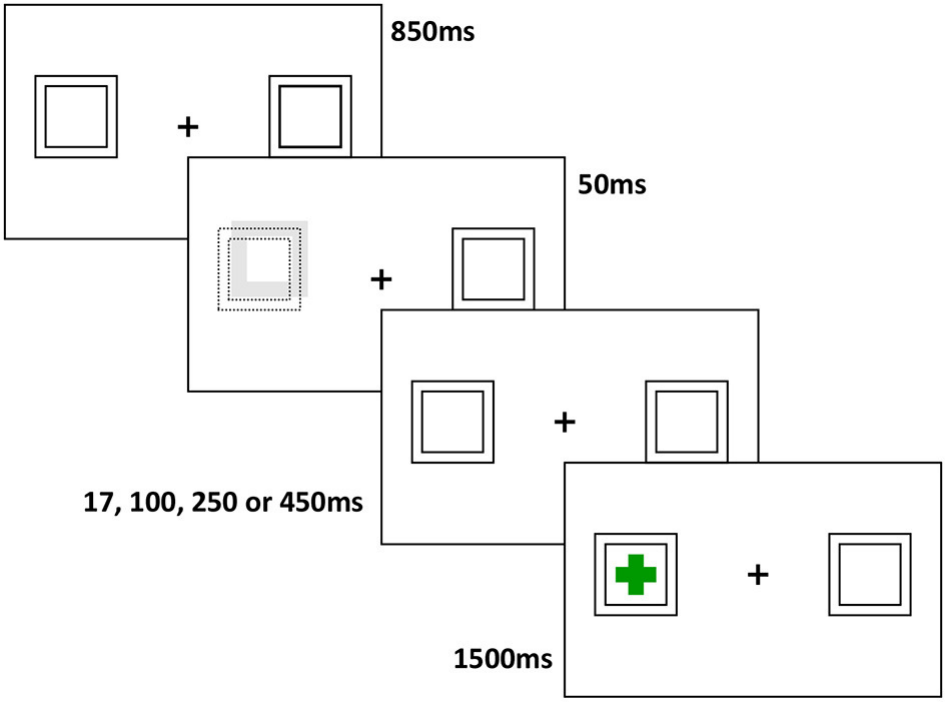
Schematic diagram of a valid trial: Following a fixation period of 850 ms, a cue is presented for 50 ms (displacement and increased luminance of one of the two peripheral boxes), followed at various intervals by a target cross in either the same, or opposite hemifield. The target remains on screen for 1,500 ms, and subjects are asked to generate a saccade to the target as quickly as possible.

The illumination of the box was not predictive of an upcoming target; on 50% of trials the green cross subsequently appeared in the illuminated box (valid trials), and on 50% of trials, the green cross appeared in the opposite box (invalid trials). A schematic diagram of a Valid trial is provided in Figure 1.

#### Data Analysis

Output from the video-oculographic system was analysed using customised software written in MATLAB. Variables of interest were error rate and saccade latency (ms). An error was defined as an eye movement exceeding 1.5 degrees in the direction of the illuminated box prior to or within 100 ms of the presentation of the target cross and calculated as proportion of total trials. Saccade latency reflected the onset of a saccade minus target presentation time. Saccade onset was determined as displacement of the eyes from central fixation, corresponding with a change in the velocity profile of the saccade trace (>30 degrees per second). Trials were removed from analysis of latency if an error was performed, fixation was not maintained within 2 degrees of the central cross or a blink occurred at target or saccade onset or no response was made.

A two-way ANOVA was conducted to compare error rates between groups, with between-subjects factor of Group (VSS vs. Controls) and within-subjects factor of SOA (67, 150, 300, and 500 ms). Eligible latency trials were submitted to a 3-way ANOVA with between-subjects factor of Group (VSS vs. Controls) and within-subjects factor of SOA (67, 150, 300, and 500 ms) and Trial Type (Valid vs. Invalid). *Post-hoc* analyses were conducted using ANOVA. Where DASS depression, stress or FSS scores significantly correlated with any experimental variable, these scores were used as a covariate.

To assess whether the onset of IOR differed between groups, planned comparisons were conducted at each SOA, with valid and invalid trial latencies compared for each Group.

Correlational analyses were conducted using either Pearson's r or Spearman's rho between OM and clinical variables.

## Results

Mean latencies and error rates for controls and VSS participants can be found in Table 3.

**Table 3:** Means and standard deviations for ocular motor task variables.

	**Controls *Mean* (SD) *n* = 20**	**VSS *Mean* (SD) *n* = 40**
**Latencies (ms)**		
Invalid SOA 67	342.93 (34.10)	361.67 (43.30)
Invalid SOA 150	330.06 (30.73)	341.75 (46.91)
Invalid SOA 300	315.37 (34.96)	322.92 (45.92)
Invalid SOA 500	290.64 (36.97)	312.77 (45.64)
Valid SOA 67	323.07 (40.73)	326.66 (37.72)
Valid SOA 150	325.52 (32.39)	326.06 (43.96)
Valid SOA 300	325.34 (36.27)	326.52 (38.40)
Valid SOA 500	314.83 (35.57)	331.85 (43.41)
**Error rate (%)**		
SOA 67	1.25 (3.27)	2.97 (5.83)
SOA 150	4.27 (6.53)	4.69 (6.44)
SOA 300	15.73 (10.06)	15.57 (12.08)
SOA 500	17.60 (13.69)	18.33 (14.50)

*SOA, Stimulus Onset Asynchrony*.

### Latency

A significant main effect of SOA was found [*F*_(2.70,156.51)_ = 22.28, *p* < 0.001, ηp = 0.28], demonstrating that, overall, latencies decreased with increasing SOA. As anticipated a significant trial x SOA interaction was found [*F*_(2.58,149.67)_ = 25.38, *p* < 0.001, ηp = 0.304]. This was due to the well-known effect of the cue in the IOR task; faster latencies were found for valid trials at shorter SOAs and faster latencies for invalid trials at longer SOAs. A significant group and Trial type interaction [*F*_(1,58)_ = 4.11, *p* = 0.047, ηp = 0.066] demonstrated significantly longer invalid trial latencies than valid trial latencies, overall, for VSS participants only (MD = 7.00, *p* = 0.012). No other effects or interactions were found.

A series of planned comparisons revealed that with 67 ms SOAs, invalid trial latencies were significantly longer than valid trial latencies for both controls (*p* < 0.001) and VSS participants (*p* < 0.001), demonstrating the facilitatory effect of the cue at this SOA. With 500 ms SOAs, valid trials latencies were significantly longer than invalid trial latencies for controls (*p* < 0.001) and VSS participants (*p* < 0.001), reflecting the IOR effect at this SOA for both groups.

**Figure 2:**
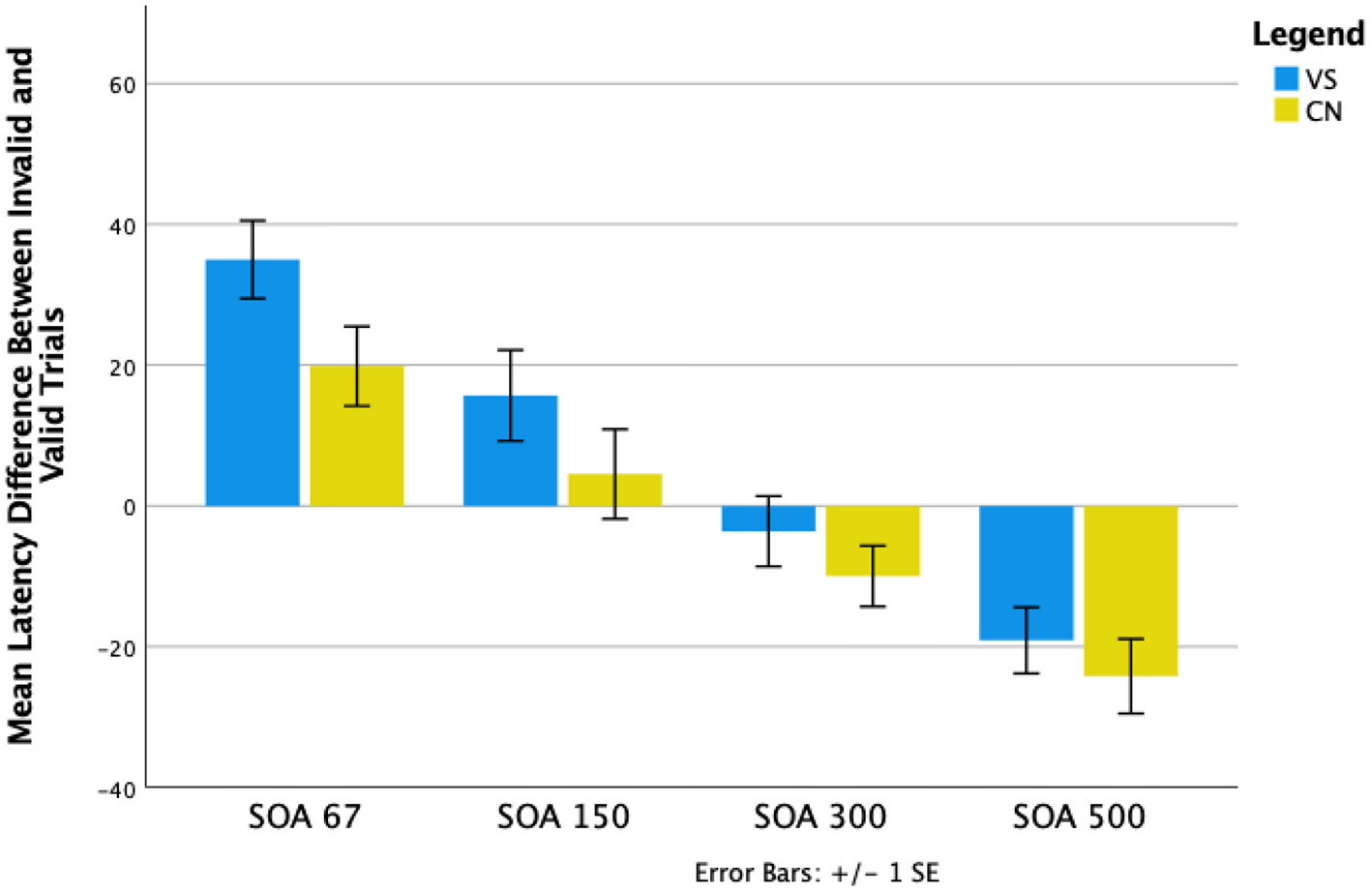
Mean differences between invalid and valid trial types across the four Stimulus Onset Asynchronies (SOAs) for VSS and control groups.

However, for controls only, there were no significant differences between trial types with 150 ms SOAs (*p* = 0.48), indicating onset of IOR at around 150 ms; for VSS participants, invalid trial latencies were still significantly longer than valid trial latencies (*p* = 0.02). IOR emerged later for VSS participants, for 300 ms SOAs, where there were no significant differences in latency between trial types (*p* = 0.48). For controls, latencies for valid trials were longer than latencies for invalid trials at this SOA (*p* = 0.03). The differences between valid and invalid trials latencies are represented in Figure 2.

### Error Rate

A significant main effect of SOA was found [*F*_(2.48,143.64)_ = 44.00, *p* < 0.001, η*p* = 0.43], demonstrating that error rate increased as SOA increased. No group effect or interaction was revealed.

### Correlations

There were no significant correlations between any OM and clinical variable after Bonferroni adjustments for multiple comparisons were applied.

## Discussion

We have previously demonstrated VSS-specific alterations to visual processing that are consistent with stronger attentional capture (8, 9). We propose that the corresponding imbalance between saccade facilitation and inhibition results in an increased number of erroneous saccades and shorter saccade latencies. Here we explicitly explored this proposal by manipulating the time course and strength of exogenous attentional capture using a classic ocular motor Posner spatial cueing paradigm, which is known to affect the temporal profile of saccade latencies. Specifically, we manipulated the time between presentation of a non-informative visual cue and a subsequent target. As expected, when the time interval between cue and target was short, saccade latencies to correctly cued locations (valid trials) were shorter than incorrectly cued locations (invalid trials) for both VSS participants and controls. Further, when the time interval between cue and target increased (to 500 ms), the cue-target relationship was inverted for both groups manifesting in IOR with saccades to invalidly cued targets generated more quickly than saccades to validly cued targets. However, the time point at which IOR occurred, differed significantly between groups. While the onset of IOR occurred at around 150ms for controls, IOR was delayed for VSS participants and was only evident at around 300 ms. This suggests a relative imbalance between facilitatory and inhibitory saccade activity in VSS participants, altering the relationship between cue and target activity. Unexpectedly, VSS participants did not generate more erroneous saccades to cue stimuli than controls.

A number of cortical and subcortical regions have been identified as important for the generation of IOR. These include the frontal and supplementary eye fields, the supramarginal gyrus, ventrolateral nucleus of the thalamus, inferior parietal lobule and the anterior cingulate cortex (24, 25), as well as networks connecting frontal and parietal regions (26, 27). These regions and tracts generate and transmit facilitatory and/or inhibitory signals regarding saccade generation, which converge and are balanced topographically within the superior colliculus (SC). The outcome is either the execution or inhibition of a saccade (28). However, when a cue is presented at the target location shortly before the visual target, there is an overlap of cue/target activity arriving at and generated within the SC that increases saccade-related activity. As activity is brought closer to threshold for release, saccade latency is also reduced (29). When a target and cue are presented in different locations, i.e., an invalid trial, there is no overlap between cue and target activity; activity decreases as the result of local inhibition within the SC (30). As a result, baseline activity is reduced when the cue appears rather than increased as seen when cue and target are in the same location, and saccades are initiated with relatively longer latencies. However, as the time between cue and target increases, the relationship between cue and target activity alters. At longer SOAs, there is no longer overlap between target and cue activity; target-aligned activity appears to be inhibited within the SC. As a result of this reduction in activity, threshold for release of a saccade for a validly cued target is delayed relative to an invalidly cued target (31).

For VSS participants, this shift from facilitation to inhibition of a saccade toward a validly cued target was delayed compared to controls, suggesting that the overlap between cue and target activity for these trials was relatively increased, resulting in greater target-aligned activity. This increase in target-aligned activity might persist longer and require less saccade-related activity to generate a response. This alteration in SC activity is likely a consequence of disruption to signals arising from other regions of the brain (28). Indeed, similar patterns of activation to that seen in the SC has been observed in the visual cortex in both human and primate studies (31, 32). In VSS, recent studies have demonstrated functional and structural alterations within the primary visual cortex (V1) (33) and ventral visual regions (34). Hypermetabolism and cortical volume increases have been reported at the intersection of the right lingual and fusiform gyri (35), and resting-state functional MRI data revealed hyperconnectivity between extrastriate and inferior temporal brain regions as well as prefrontal and parietal regions (11). While it is not possible to determine the source (cortical/subcortical location) or mechanism (increased facilitation and/or reduced inhibition) of the proposed increase in activity, we do not believe that it is being driven by frontally mediated changes altering inhibitory activity. As indicated earlier, delayed IOR onset has been reported in patients with schizophrenia (15, 36, 37). However, these individuals also tend to make more errors than healthy controls, unlike our VSS group. Researchers have attributed this to the pathological changes observed in the frontal cortex of participants with schizophrenia (38, 39), which disrupts the inhibition of irrelevant responses (40, 41). Given our prior findings of a speeded visually guided response and lack of deficit with respect to frontally mediated task-switching, cueing and Simon effects (8, 9), we suggest that this is not the case with VSS participants. Instead, we propose that the differences found here with respect to the time-course of IOR are likely due to enhanced early facilitation of saccade-related activity as a consequence of altered activation within early visual processing regions of the brain and/or disruption to thalamocortical networks.

While the pathophysiology underlying VSS is unclear, a commonly described consequence appears to be that of cortical hyperexcitability within, and beyond, the brain's visual processing regions (4, 35, 42, 43). While the SC is not directly implicated in VSS pathology, it receives input from areas previously described, including the visual cortex, frontal eye fields, and parietal cortex (44, 45). Consistent with our results here and in previous research, increased excitability within the visual cortex might increase SC activity to both the cue and target via these projections; resulting in greater and longer overlap in cue and target-aligned activity within the SC. Persistence, a consequence of this overlap, would present behaviourally as a stronger capture of attention and interruption to the onset of IOR, as was seen in our participants with VSS.

With respect to the unexpected finding in VSS that these individuals did not generate more erroneous saccades to cue stimuli than controls, it is conceivable that this reflects the relevance of the cue stimulus. Unlike our previous studies (8, 9), the visual cue used here bears no relationship with the required response. It does not *predict* the location of the up-coming target, as does the cue in the endogenously cued saccade paradigm (9), and it does not provide information about where a person *should* look as in the visually guided saccade paradigm or directive stimuli used in the antisaccade (8) or Simon effect paradigms (9). In short, it is likely to engender less attentional capture than these previously used visual stimuli. While we demonstrate here what is likely to be enhanced facilitation by the cue, the increased level of activation of saccade-related neurons does not appear to exceed threshold for release, hence no more errors to cue stimuli than controls.

These findings, demonstrating differences in VSS in the temporal relationship between competing sensory events that manipulate facilitatory and inhibitory processes are consistent with our proposal that shifts of attention more strongly increase saccade-related activity in VSS. These changes conceivably reflect changes within thalamo-cortical processing networks, in particular attentional networks. This is the first study to assess temporal changes in allocation of visuospatial attention in VSS and provides a more refined saccadic behavioural profile of VSS that can be interrogated using sophisticated neuroimaging techniques and may, in combination with other saccadic markers, be used to monitor the efficacy of any future treatments.

## Data Availability Statement

The raw data supporting the conclusions of this article will be made available by the authors, without undue reservation.

## Ethics Statement

Ethical approval was granted by the Monash University Human Research Ethics Committee. All participants provided written informed consent prior to participation in the study in accordance with the declaration of Helsinki.

## Author Contributions

Acquisition of data was handled by PF and ES. PF wrote the first draught of the manuscript. JF, MC, and OW supervised the project. All authors critically revised the manuscript and approved the submitted version.

## Conflict of Interest

The authors declare that the research was conducted in the absence of any commercial or financial relationships that could be construed as a potential conflict of interest.

## Publisher's Note

All claims expressed in this article are solely those of the authors and do not necessarily represent those of their affiliated organizations, or those of the publisher, the editors and the reviewers. Any product that may be evaluated in this article, or claim that may be made by its manufacturer, is not guaranteed or endorsed by the publisher.
